# Optimizing Whole-Cell Biosensors for the Early Detection of Crop Infections: A Proof-of-Concept Study

**DOI:** 10.3390/bios15050300

**Published:** 2025-05-08

**Authors:** Nadav Zanger, Evgeni Eltzov

**Affiliations:** 1 Institute of Postharvest and Food Science, Department of Postharvest Science, Volcani Institute, Agricultural Research Organization, Rishon LeZion 7505101, Israel; nadav.zanger@mail.huji.ac.il; 2 Institute of Biochemistry, Food Science and Nutrition, Faculty of Agriculture, Food and Environment, The Hebrew University of Jerusalem, Rehovot 76100, Israel

**Keywords:** whole-cell-based biosensor, bioluminescence, postharvest crops monitoring, bacterial immobilization, volatile organic compounds (VOCs), real-time monitoring

## Abstract

This study presents a proof-of-concept evaluation of optimized whole-cell biosensors designed for the real-time detection of crop infections. Genetically engineered luminescent bacterial strains were used to detect volatile organic compounds (VOCs) emitted by crops during spoilage. Key factors investigated include bacterial uniformity, nutrient supply, and temperature effects. The results demonstrated that lower temperatures (+4 °C) yielded higher sensor sensitivity and prolonged bacterial viability. A proof-of-concept evaluation was conducted in storage-like conditions, showing effective infection detection in potatoes. These findings underscore the potential of whole-cell-based biosensors for monitoring postharvest production in cold storage environments.

## 1. Introduction

Food loss is a significant global issue with substantial economic and environmental impacts [1]. A major contributing factor to food loss is the spoilage of crops during storage and transportation. One promising approach to mitigating this problem is the early detection of decay processes through the monitoring of volatile organic compounds (VOCs) emitted by crops. Changes in VOC profile can indicate the beginning of spoilage, allowing for timely interventions to prevent significant losses [2].

Metabolism is integral to the proper functioning of any organism, involving a series of chemical and enzymatic reactions that occur both at the cellular level and throughout the entire organism. This process encompasses the absorption of substances from the environment, their processing, energy extraction, and waste elimination, including the release of gases and specific molecules [3]. Among them, VOCs emitted by plants serve as indicators of plant health, playing crucial roles in growth, survival, communication, defense, and signaling against pests and diseases [4]. Notably, the types and quantities of VOCs emitted by plants vary across different physiological conditions and metabolic stages, providing insights into specific physiological conditions when analyzed comprehensively [5].

Traditional methods of VOC detection, primarily based on gas chromatography, are complex, non-portable, and incapable of continuous monitoring [6]. Therefore, there is a pressing need for alternative monitoring techniques that are sensitive, capable of real-time and continuous operation, and able to assess the complete VOC profile in storage rooms or during shipment. Conventional gas chromatography–mass spectrometry (GC-MS) methods, while effective in identifying volatile compounds post-pathogenic infection, are cumbersome, time-consuming, and lack the durability for continuous VOC monitoring in storage or transportation facilities and enclosed spaces around packaged goods [7].

A novel solution to these challenges is the development of biosensors that detect specific VOCs. These biosensors may be operated by recognizing target compounds through specific entities, such as antibodies or enzymes, or by monitoring physical changes in response to VOC exposure [8]. Whole-cell biosensors, which utilize entities like bacteria exposed to certain molecules, offer a promising approach to monitoring biological processes through changes in bacterial cells [2,9,10]. A critical aspect of developing such biosensors is employing a robust technique for immobilizing bacteria and a sensor capable of monitoring changes in the immobilized bacteria.

Previous research has demonstrated the potential of a whole-cell biosensor based on genetically modified bioluminescent bioreporters immobilized within hydrogel matrices. This biosensor successfully detected disease processes in agricultural crops, such as potatoes and citrus fruits, infected with pathogens [2,9,10]. The biosensor system, consisting of an alginate-based hydrogel embedding the bacteria along with a luminescence-detecting sensor, showed significant potential for early-stage disease detection in crops. A schematic overview of the proposed biosensor system is provided in the Supplementary Materials (Figure S1) to illustrate the detection concept, including air sampling, bioreporter activation, and signal output.

Despite the promise of hydrogels containing bioreporter bacteria as an economical and straightforward method for detecting crop-emitted VOCs, several challenges remain. Primarily, the viability of bacteria within hydrogels must be maintained over extended periods and under various conditions to ensure the reliability of the biosensor. While previous studies have used ideal conditions for hydrogel preparation and bacterial survival, the resilience of whole-cell biosensor systems under realistic storage and shipment conditions remains uncertain. This study aims to optimize the immobilization approach for bioluminescent bacteria in calcium alginate hydrogels to enhance VOC detection in agricultural applications. Specifically, genetically engineered luminescent bioreporter bacterial strains will be used to detect volatile markers strongly induced by pathogens in crops such as potatoes and citrus fruits. The study will simulate various conditions to evaluate the biosensor’s ability to withstand real-world scenarios, thereby improving its reliability and accuracy in monitoring crop health.

## 2. Materials and Methods

### 2.1. Materials

White opaque 96-well microtiter plates (30396) were purchased from SPL Life Sciences (Naechon-Myeon, Pocheon-si, Republic of Korea). Luria–Bertani broth (LB) (LBX1L) and Terrific Broth (TB) (TRB0102) were purchased from Formedium Ltd. (Norfolk, UK). Ampicillin sodium salt (BP1760-25) was purchased from Fisher BioReagents (Pittsburgh, PA, USA). Alginic acid sodium salt (B25266) was obtained from Alfa Aesar (Lancashire, UK). Calcium chloride (A119.2) was purchased from Carl Roth GmbH (Karlsruhe, Germany). Plastic syringes (7290010993659) were purchased from Kalir (Petah-Tikva, Israel). All stock solutions were diluted with double-distilled water and stored at temperatures suggested by the manufacturers.

### 2.2. Equipment

An incubator and rotary shaker, BEING THZ-100 (EMIN Myanmar Co., Ltd., Yangon, Myanmar), were used to grow a bacterial panel. A GENESYS^TM^ 10S UV-Visible spectrophotometer (Thermo Scientific, Yangon, MA, USA) was used to measure the absorbance to adjust the optical density of the bacterial panel culture. A SynergyHTX multi-mode reader (BioTek Instruments Inc., Winooski, VT, Canada) was used to measure the bioluminescent signal of the bacterial panel. The IVIS Lumina LT In Vivo Imaging System was used to capture the bioluminescent activity of the bacterial tablets.

### 2.3. Bacterial Strains and Growth Conditions

Two different *Escherichia coli* strains were used in this study: TV1061, obtained from S. Belkin (Hebrew University, Jerusalem, Israel), and a homemade strain, *DnaK*. Both strains contained a plasmid with a promoter fused to the lux CDABE operon using a multi-copy plasmid (mcp), responsible for synthesizing luciferase and its substrates. TV1061 (grpE) responds to cytotoxic substances, while *DnaK* senses molecular stress from misfolded proteins. The bacterial stocks, stored at −80 °C in 20% (*v*/*v*) glycerol, were streaked on LB-agar plates (10 g/L tryptone, 5 g/L yeast extract, and 5 g/L NaCl) supplemented with 100 μg/mL ampicillin and incubated for 24 h at 37 °C. The strains were grown in 10 mL LB medium with 100 μg/mL ampicillin overnight at 37 °C on a 200 rpm rotary shaker. The following day, the bacterial cultures were diluted to approximately 10^7^ cells/mL and regrown without antibiotics to the early exponential phase (OD600 nm = 0.2). A strain of *Pectobacterium brasiliense* (Pbr) used in the current study was identified and characterized using several molecular methods, namely PCR, RT-PCR, and dnaX or gapA sequencing [11]. The Pbr inoculum was prepared by growing the pathogen in LB (without antibiotics) to an OD_600nm_ = 0.6.

### 2.4. Inoculation of Whole Tubers with Pectobacterium Brasiliense

Infected and non-infected potato samples were prepared using tubers (*n* = 3–5) sourced from a commercial packhouse. Each potato was nicked in multiple locations, and 45 μL of Pbr pathogen was applied to infect the samples.

### 2.5. Preparation of Calcium Alginate Tablets for Bacterial Immobilization

The calcium alginate tablets were prepared as described previously [12]. First, cellulose filter paper was cut into rectangles (10 cm × 7 cm), rolled into tubes (6 mm in diameter), and sealed at the bottom using the cap of a 500 μL centrifuge tube. The harvested bacterial cells were mixed 1:1 with 2.5% (*w*/*v*) sodium alginate solution and loaded into the cellulose tubes with a 10 mL syringe. After 5 min to remove air bubbles, the mixture underwent 20 min polymerization in 0.25 M calcium chloride. The alginate tubes were then removed from the cellulose and cut into 3 mm tablets using a customized 3D-printed holder.

### 2.6. Evaluation of Effect of the Medium on the Cells’ Viability and Functionality

To evaluate the effect of different media on sensor viability, bioreporter cells were grown overnight in 10 mL LB medium with 100 μg/mL ampicillin at 37 °C. The following day, the culture was diluted to approximately 10^7^ cells/mL and regrown without antibiotics to the early exponential phase (OD600 nm = 0.2). After centrifugation, the LB media was replaced with various nutrient media as follows: Minimum Salt (K_2_HPO_4_ 1.73 g/L, KH_2_PO_4_ 0.68 g/L, MgSO_4_ 0.1 g/L, FeSO_4_ 0.03 g/L, NH_4_NO_3_ 1.0 g/L, CaCl_2_ 0.02 g/L, and NaCl 4.0 g/L), SOC (dextrose 3.6 g/L, KCl 0.186 g/L, MgSO_4_ 4.8 g/L, tryptone 20 g/L, and yeast extract 5 g/L), yeast extract (5 g/L), Terrific Broth (tryptone 12 g/L, yeast extract 24 g/L, and glycerin 2 mL), and tryptone (10 g/L). The cultures were then placed in a 96-well plate, divided into two sets (with and without 2% ethanol), sealed, and continuously monitored at 27 °C for 72 h using a SynergyHTX multi-mode plate reader (BioTek Instruments Inc., Winooski, VT, USA) to measure bioluminescent responses and cell viability (OD600 nm).

### 2.7. Evaluation Impact of Polymerization Processes on Bacterial Uniformity in the Membrane

For the evaluation of the polymerization process, cellulose filter paper tubes were rolled into 6 mm diameter cylinders. These cylinders were placed in a 100 mL beaker and filled with a mixture of harvested bacterial cells, 2.5% (*w*/*v*) sodium alginate solution, and 2% (*v*/*v*) ethanol for bioluminescence activation. The beaker was then filled with 0.25 M calcium chloride solution to initiate polymerization. The entire setup was placed in an IVIS imaging system to assess bacterial uniformity within the alginate matrix.

### 2.8. Evaluation of the Effect of Storage Temperature and Nutrient Supply on Bacterial Viability and Activity

Calcium alginate tablets were prepared as previously described and stored in 96-well plates under different temperatures (e.g., +4 °C, +10 °C, and +25 °C). For each temperature, two sets of tablets were used: one received nutrients during the evaluation, whereas the other did not. At each time point, the surrounding fluids were extracted and replaced with 15 μL of 2% ethanol solution. Light intensity was measured for 2 h, and then one set of tablets received 30 μL of fresh LB broth while the other remained dry. In this experiment, 2% (*v*/*v*) ethanol was not added to all samples simultaneously. Instead, ethanol was introduced to separate groups of tablets on different days: on Day 0, Day 1, Day 2, Day 7, and Day 14. Until ethanol was added, the samples received only regular medium exchange (with or without LB). For example, in the ‘14-D’ group, ethanol was added only after 14 days of incubation. This design allowed for the evaluation of bacterial responsiveness to ethanol following different storage durations and nutrient conditions. These testing groups are labeled in the figure as ‘0-D’, ‘1-D’, …, and ‘14-D’, indicating the number of days before ethanol exposure.

### 2.9. Assessing the Effect of Environmental Temperature on Whole-Cell-Based Sensor Functionality

To assess the effect of temperature on whole-cell-based sensor functionality, calcium alginate tablets containing the TV1061 strain were prepared as outlined in Section 2.5. The tablets were positioned on top of a CMOS-based device (Anitoa 4-Channel ULS24 Solution Kit, Menlo Park, CA, USA) and then exposed to samples with and without 2% (*v*/*v*) ethanol. The system was incubated in a closed glass jar for 10 h at varying temperatures (+4 °C, +10 °C, and +25 °C). Bacterial bioluminescence activity, expressed as Relative Light Units (RLUs), was recorded using ULVision software (V2.0, Anitoa, Menlo Park, CA, USA).

### 2.10. Temperature and Distance Impact on Bioreporters’ Response Times for the Detection of Pectobacterium Brasiliense in Potatoes

The temperature and distance impact on the bioreporters’ response times were tested by using the responses of the *DanK* bacterial strain to infected potato tubers with soft rot disease. The inoculation of the potato tubers with *Pactobacterium brasiliense* was followed as previously explained and described in a recent paper by the research group [2]. Thereafter, the bioreporter strain containing genetically modified bacteria sensitive to the presence of *Pbr* (*DnaK*) was immobilized in alginate tablets. The potatoes, infected and non-infected whole tubers (*n* = 3 of each), were kept in a different isolated box with separate isolated boxes along with the tablets. The tablets were placed at different distances from the potatoes (5, 10, 15, and 20 cm). The boxes were kept at different temperatures (+4 °C, +10 °C, and +25 °C) to evaluate the effects of different conditions on the tablets that were exposed to infected potato tubers for 2 h. Lastly, the alginate tablets were placed in 96-well plates for the measurement of the bioluminescence activity of the bioreporters every 30 min in a SynergyHTX multi-mode reader (BioTek).

### 2.11. Assessing the Whole Cells’ Sensitivity

To evaluate the sensitivity of the *E. coli DnaK* bioreporter strain, calcium alginate-immobilized bioreporter cells were exposed to a series of 2-Phenylethyl Alcohol (2-PEA) concentrations ranging from 54,000 ppb to 0.000054 ppb. For each concentration, bioreporter tablets were placed in one well of a 96-well white microtiter plate, while 10 μL of the corresponding 2-PEA solution was deposited in an adjacent well. Prior to cell deposition, a small channel was drilled between these two wells to allow for the diffusion of volatile compounds. After sample preparation, the plate was sealed with a cover, ensuring that the exposure of the bioreporter cells to 2-PEA occurred exclusively through vapor-phase diffusion rather than direct contact with liquid. Each experiment included *n* = 8 repeats for each concentration, and a minimum of 10 independent experiments were conducted to ensure statistical robustness. The measurement temperature was maintained at 26 °C, and bioluminescence signals were recorded every 5 min for 10 h using a Synergy HTX Multi-Mode Reader (BioTek Instruments, Winooski, VT, USA). Statistical analysis was performed using a paired two-sample *t*-test (*p* < 0.05) to determine significant differences in bioluminescent responses.

### 2.12. Assessing the Capabilities of the Whole Cell-Based Sensor for the Determination of Infection in the Stored Tubers

To evaluate the sensor’s ability to detect *Pectobacterium brasiliense* in potato tubers, the *DnaK* strain was immobilized in calcium alginate tablets, positioned on top of a CMOS sensor, and placed near both infected and non-infected tubers (infected as described in Section 2.4) in an open box at +4 °C. The bioreporter cells were exposed to VOCs emitted by the samples for 11 h. During this period, bacterial bioluminescence activity, expressed as Relative Light Units (RLUs), was continuously measured using the CMOS-based device and recorded through ULVision software.

### 2.13. Data Analysis

The bioluminescence signal indicative of the bacterial panel responses is expressed as an induction factor (IF), which was calculated using the formula: IF = Bs/Bc, where Bs is the maximum bioluminescence signal of the tested sample, and Bc is the maximum bioluminescence signal of the control.

## 3. Results and Discussion

### 3.1. Evaluation of the Effect of Polymerization Processes on Bacterial Uniformity

**Figure 1:**
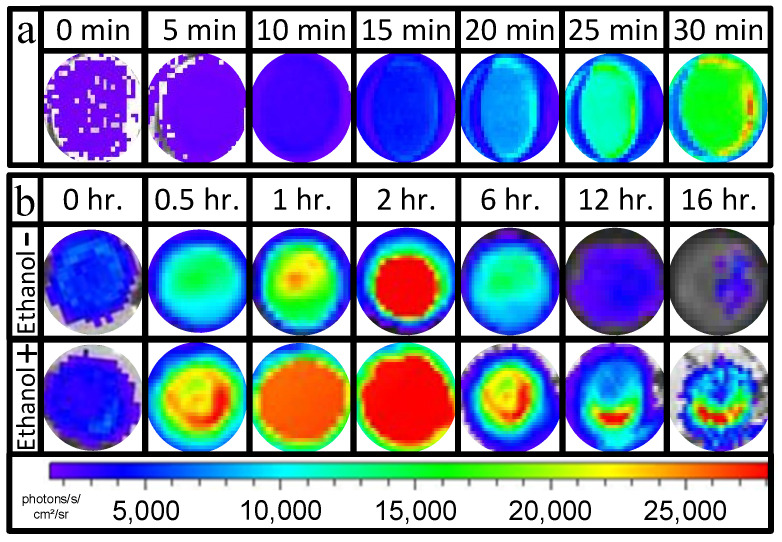
Effect of calcium alginate polymerization processes on bacterial TV1061 strain uniformity in the membrane during matrix formation (**a**) and in the further responses to 2% (*v*/*v*) ethanol (**b**).

In this section, the effect of the calcium alginate polymerization processes on bacterial uniformity is evaluated. Through this assessment, we aimed to understand how polymerization processes influence the distribution and consistency of bacteria within the hydrogel matrix, which is critical for ensuring the reliability and performance of the biosensor. At the starting point, we took conditions that were tested in a previous study, which are optimized immobilization processes to ensure maximum uniformity through tablet formation [12]. This approach allowed us to establish a baseline for evaluating how variations in calcium alginate polymerization processes might impact the bacterial distribution and consistency within the hydrogel matrix. Figure 1a demonstrates the effect of the polymerization processes on bacterial diffusion through the immobilization processes. For this, bacteria were mixed with an alginate solution and ethanol to induce bacterial responses. Then, the induced bacteria were added to the calcium solution, which allowed for the determination of their position by evaluating the light intensity at different positions within the tablet. Figure 1a demonstrates that higher light activities were observed on the edges of the tablets, suggesting a higher cell concentration in those areas. The possible reason for this phenomenon is that during polymerization, water molecules are replaced by calcium ions, causing water to be expelled from the internal area to the outside. This migration can produce forces that pull bacteria from the internal area to the tablet borders. Moreover, part of the bacteria may also be expelled from the tablets due to this process, thereby reducing their concentration within the tablets. Such processes will induce variance in the tablet’s response because this uncontrolled migration will randomly reduce bacterial concentrations, resulting in tablets with different bacterial concentrations that respond variably to the same constant conditions. Similar difficulties in the formation of alginate membranes with uniform and reproducible content have been reported previously [13]. To demonstrate this effect, bacteria immobilized in the tablets were exposed to 2% (*v*/*v*) ethanol and compared to the untreated matrices. Figure 1b not only visualizes the inducing effect of ethanol on immobilized cells, whereby bacteria exposed to ethanol produced stronger light activities and longer inducing effects compared to untreated cells, but also demonstrates non-uniform light activity in all tested membranes. In both cases, stronger light intensity was observed at the tablet borders, suggesting higher cell concentrations in these areas. Such processes will induce variance in the tablet’s response because this uncontrolled migration will randomly reduce bacterial concentrations, resulting in tablets with different bacterial concentrations that respond variably to the same constant conditions. To address the issue of nonuniform bacterial distribution within calcium alginate tablets, several strategies can be considered to modify the polymerization process and enhance the consistency of bacterial immobilization. Optimizing polymerization conditions, such as the concentration of calcium ions and the rate of gelation, can help control the diffusion of components within the alginate matrix, minimizing the forces that cause their migration [14]. Incorporating additives into the alginate solution that interact with the bacteria or the alginate network can stabilize bacterial positions within the matrix. For instance, the use of biocompatible polymers (e.g., chitosan) has been shown to reduce bacterial mobility and enhance uniformity [15]. Employing advanced pre-encapsulation mixing techniques, such as microfluidic-based applications, can also ensure a more even distribution of immobilized components in the alginate solution before gelation [16]. Implementing a layer-by-layer encapsulation approach may achieve uniform distribution by gradually adding alginate (with or without additives) and bacteria in successive layers [17]. By implementing these strategies, the nonuniformity in bacterial concentrations within calcium alginate tablets can be mitigated, significantly enhancing the reliability and performance of the biosensor and leading to more accurate and reproducible detection of volatile organic compounds under various environmental conditions.

### 3.2. Effect of the Nutrient Source on Bacterial Viability and Activity

**Figure 2:**
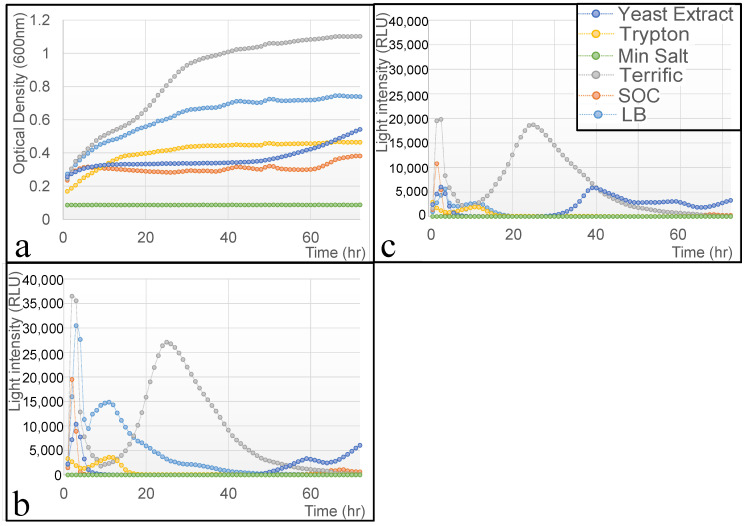
Effect of different medium types on bacterial growth (TV1061) patterns (**a**) and bioluminescent activity in response to solutions with (**b**) and without (**c**) 2% (*v*/*v*) ethanol addition.

Real-time whole-cell-based biosensors rely on genetically modified bacteria immobilized within a matrix, continuously exposed to environmental conditions to generate a measurable, dose-dependent signal in response to a target analyte. However, these systems face a fundamental challenge: nutrient depletion. Unlike bioreactor-based designs that continuously replenish nutrients [18,19] or systems where fresh media are added to a water stream [20,21], biosensors designed for air-based applications have a non-renewable nutrient supply. In such settings, bacteria consume available nutrients over time, eventually ceasing activity once all resources are exhausted. This necessitates careful selection of media that sustain bacterial viability while maintaining biosensor responsiveness over extended periods. To address this issue, the effect of six different nutrient media (e.g., Luria–Bertani (LB), SOC, Terrific Broth, tryptone, yeast extract, and Minimal Salt) on bacterial growth and response efficiency was evaluated. The ideal medium should balance bacterial longevity with specific and stable bioluminescent activity without interfering with sensor function. Figure 2 presents the growth dynamics (Figure 2a) and bioluminescent responses (Figure 2b,c) of bacteria exposed to 2% (*v*/*v*) ethanol across different media compositions. Terrific Broth exhibited the highest bacterial growth rate, even surpassing LB, the standard medium for *E. coli* cultivation. This can be attributed to its rich composition, which includes tryptone, yeast extract, glycerol, and a phosphate buffer (Table 1). However, Terrific Broth also induced high background luminescence, likely due to glycerol metabolism, making it unsuitable for biosensor applications [22,23,24]. LB provided a balanced environment, sustaining moderate bacterial growth while maintaining a selective, ethanol-induced bioluminescent response, making it the most promising candidate. SOC medium initially promoted rapid growth, but its high glucose and magnesium salt content likely induced osmotic stress, leading to early suppression of bioluminescent responses. Tryptone and yeast extract alone provided limited bacterial support, with yeast extract showing delayed bioluminescence recovery. Minimal Salt completely failed to support bacterial viability or sensor function, confirming its inadequacy for biosensor applications. These findings suggest that LB is the optimal medium, providing a balance between bacterial longevity, stable sensor function, and selective response capabilities. Selecting an appropriate nutrient source is crucial for ensuring prolonged biosensor activity, enhancing the system’s practical application in the real-time monitoring of volatile organic compounds (VOCs).

### 3.3. Effect of Temperature and Nutrient Supply on Bacterial Functionality

**Figure 3:**
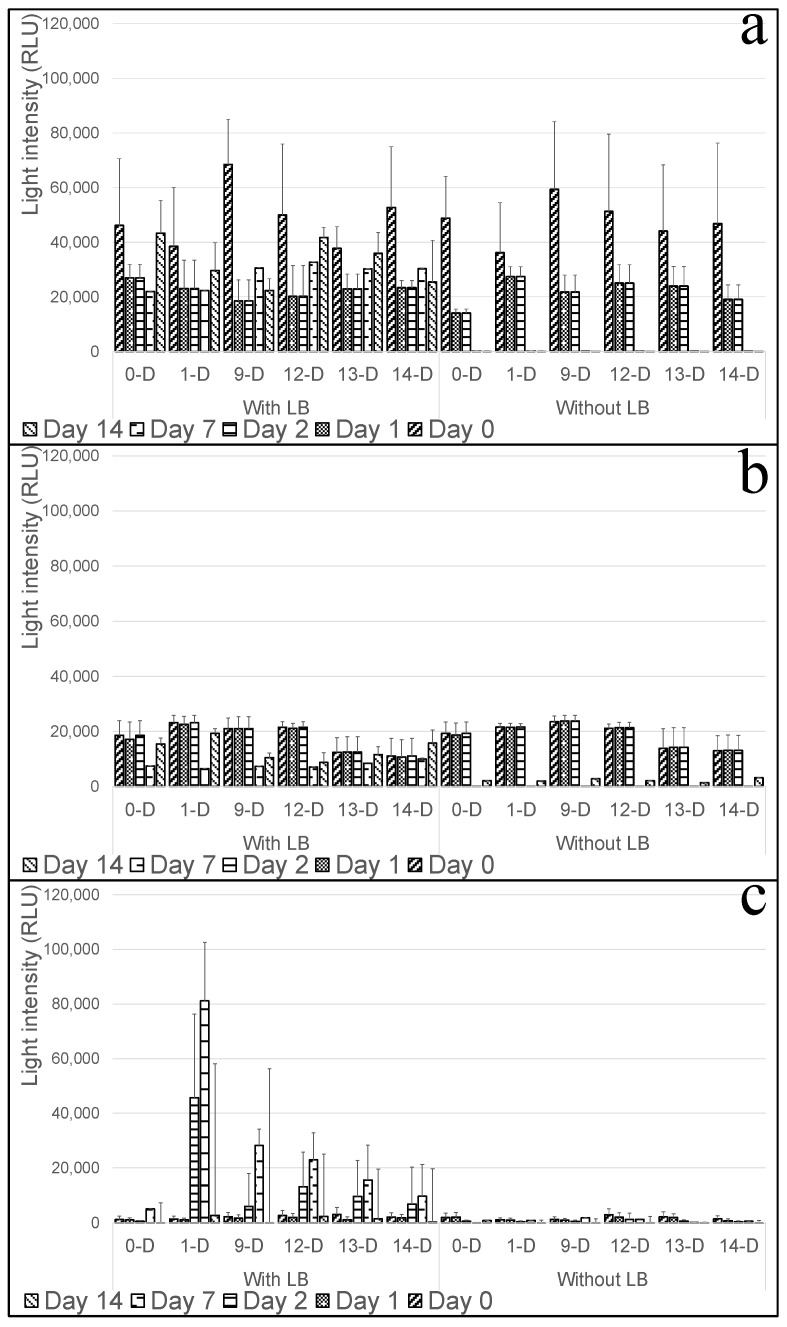
Bioluminescent response of the bacterial TV1061 strain immobilized in calcium alginate tablets under three storage temperatures: (**a**) +4 °C, (**b**) +10 °C, and (**c**) +25 °C. For each temperature, the tablets were either supplemented with LB or not. Ethanol was introduced at different time points (Day 0, 1, 2, 7, or 14) to test sensor responsiveness after varied incubation periods. ‘X-D’ indicates that ethanol was added on Day X after daily medium exchange. Peak light intensity was measured for each condition (*n* = 3, mean ± SD).

The viability and response of bioluminescent bacterial reporters depend on external factors such as nutrient availability and environmental temperature, which influence metabolic activity and overall sensor performance [25,26]. In this study, we examined the impact of nutrient supplementation (LB medium) and incubation temperatures (+4 °C, +10 °C, +25 °C) on bacterial responsiveness to 2% (*w*/*v*) ethanol over a 14-day period (Figure 3). In this experiment, 2% (*v*/*v*) ethanol was not introduced to all samples at once. Instead, separate sets of tablets were exposed to ethanol after varying incubation periods—on Day 0, Day 1, Day 2, Day 7, and Day 14—while receiving either fresh LB or no supplementation. This approach allowed us to monitor the longevity and functionality of bacteria stored under different conditions and tested at different time points, as shown in Figure 3. Without LB supplementation, bacterial cells did not survive beyond three days at any temperature, leading to a complete loss of bioluminescence signals. However, at +4 °C and +10 °C, some initial light activity was observed even in the absence of nutrients, suggesting that the slower metabolic rate at lower temperatures extended bacterial viability by reducing nutrient depletion. Indeed, at +4 °C (Figure 3a), the bioluminescent response was significantly higher than at +10 °C (Figure 3b), likely due to reduced metabolic activity enabling ethanol accumulation and enhancing its toxic effect on the bacteria [25,26]. In contrast, at +25 °C (Figure 3c), minimal responses were detected without nutrient supplementation. The addition of LB medium sustained bacterial activity throughout the entire experiment at all temperatures, but the strongest responses were recorded at +4 °C, supporting the hypothesis that at lower temperatures, slower metabolism delays the depletion of available nutrients while maintaining bacterial functionality. A different response pattern was observed at +25 °C, whereby strong induction peaks appeared when LB was added on days 2 and 7, but rapid signal decay followed. The likely explanation is that at higher temperatures, accelerated bacterial growth leads to faster nutrient depletion, inducing physiological stress that triggers biofilm formation as a survival strategy [27]. These findings emphasize the necessity of an optimized nutrient supply system for maintaining bacterial viability in long-term biosensor applications. For future biosensor development, integrating a controlled LB replenishment system could enhance sustained sensor performance, ensuring reliable and continuous detection of volatile analytes in air and water environments.

### 3.4. Effect of Temperature on Bioreporter Response and Sensor Performance

**Figure 4:**
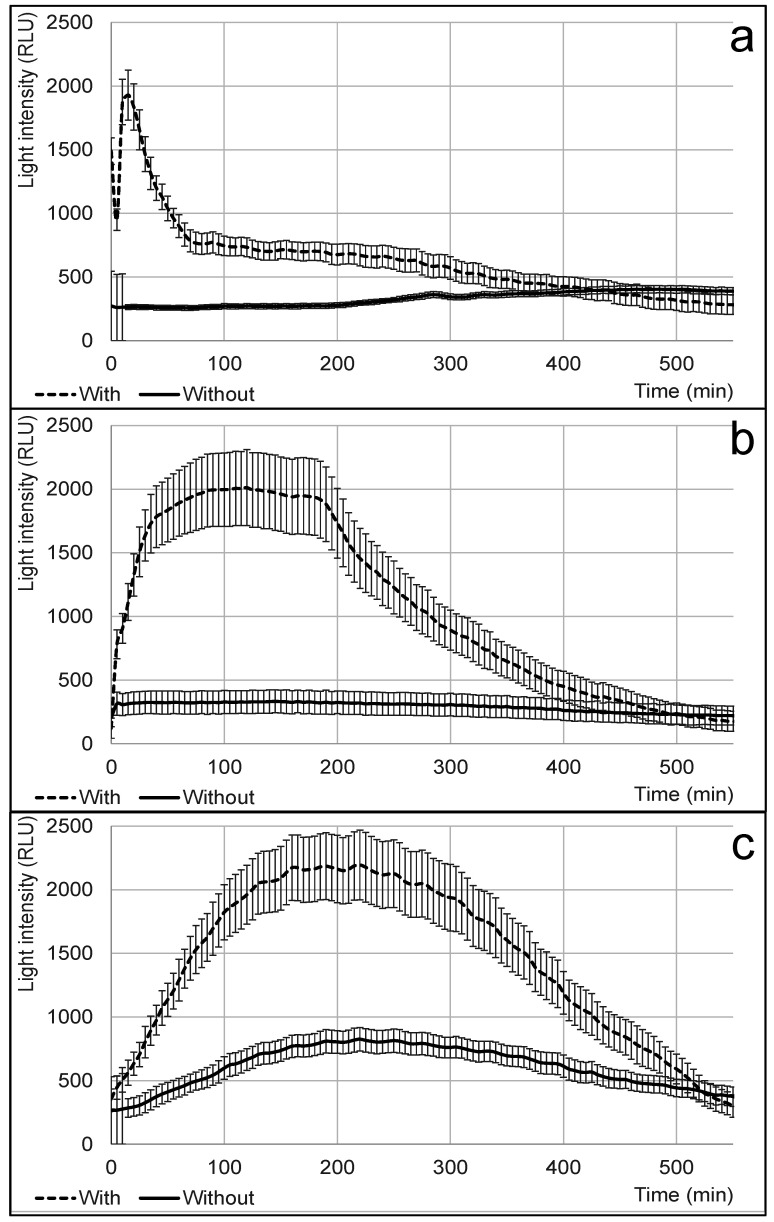
Effect of the different temperatures (+25 °C (**a**), +10 °C (**b**), and +4 °C (**c**)) on TV1061 strain (sensitive to the cytotoxic stresses) functionality. Cell activity was tested by exposing bacteria to 2% (*v*/*v*) ethanol (with) and pure water (without).

Temperature is a critical factor influencing biosensor performance, particularly in whole-cell-based applications in which bacterial metabolism dictates response times and signal intensity. In this study, we evaluated the effect of different temperatures (+4 °C, +10 °C, and +25 °C) on the bioluminescent response of the TV1061 strain, a cytotoxic stress-sensitive bioreporter, to ethanol exposure over a 10 h period. The results (Figure 4) indicate that the sensor remained functional across all tested temperatures, with ethanol successfully inducing luminescence under each condition. However, a clear temperature-dependent delay in response time was observed, with bacteria at +25 °C responding within 30 min, while those at +10 °C and +4 °C exhibited delayed responses at 100 and 200 min, respectively. This delay is likely due to the reduced metabolic rate and enzymatic activity at lower temperatures, slowing the biochemical processes required for bioluminescence generation. Interestingly, at +4 °C, a minor induction effect was observed even in control samples (Figure 4c, “without ethanol”), suggesting that the *grpE::lux* bioreporter may also respond to cold stress, potentially due to protein misfolding that triggers stress-related gene expression. While the maximum luminescence intensities were comparable across temperatures, the duration and pattern of the response varied, indicating that environmental conditions influence the sustained activity of the biosensor. These findings highlight the importance of optimizing detection parameters when deploying biosensors in cold storage environments. While lower temperatures can enhance bacterial viability over extended periods, they also slow sensor responses, which may affect real-time monitoring applications for postharvest produce.

### 3.5. Assessment of Temperature’s Impact on Bioreporters’ Response Times for the Detection of Pectobacterium in Potatoes

**Figure 5:**
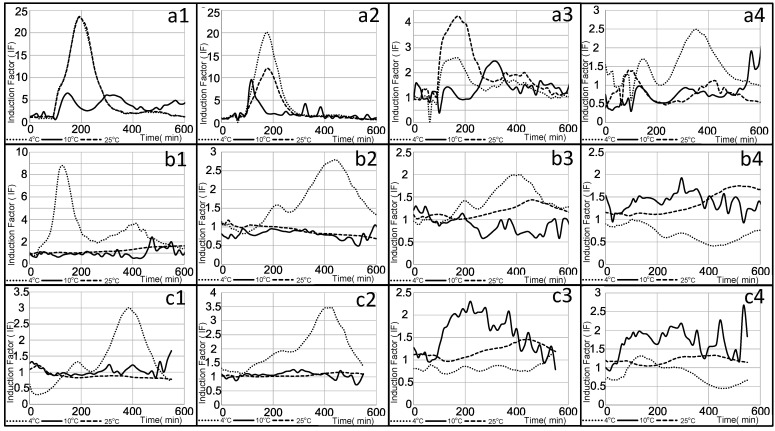
Effect of the distance from the infection source on the *DnaK* strain response at different measurement temperatures (e.g., +4 °C, +10 °C, and +25 °C). The measurement was taken at (**a**) 30, (**b**) 90, and (**c**) 120 min after exposure to the infection. (**1**, **2**, **3**, and **4**) represent distances of 5 cm, 10 cm, 15 cm, and 20 cm from the infection source, respectively.

In this phase, genetically modified bacteria (i.e., the *DnaK* strain) sensitive to the presence of *Pectobacterium brasiliense* infection in potatoes were utilized. These bacteria were engineered in a previous study to enhance their ability to detect specific volatile compounds released during the early stages of *Pbr* infection. Their ability to identify infection in potato tubers has been demonstrated, making them ideal candidates for assessing the impact of environmental factors, such as temperature and distance, on biosensor efficiency and response time in detecting crop diseases. Immobilized bacteria were placed at different distances (e.g., 5, 10, 15, and 20 cm) from the infection source (i.e., infected potato) in the box and measured at different times. The effect of temperature on such responses was also evaluated. Similar to the previous results with the general strain, the strongest responses were observed at the lowest tested temperature (+4 °C). Figure 5 demonstrates a correlation between distance and response time. The diffusion of VOCs from the infection source has a stronger impact on bioreporters positioned farther from the source than those closer, with clear dose-dependent responses observed. At +4 °C, 30 min after exposure, bioreporters located 5 cm from the infection showed a decrease in the induction effect (as induction factor values) from 24 to 20, 4.1, and 2.5 at distances of 10, 15, and 20 cm, respectively (Figure 5(a1–a4)). This dose-dependent trend persisted throughout the experiment. For the +4 °C incubation temperature, at 90 min, inhibition was observed at 20 cm, and by 120 min, both 15 and 20 cm distances showed inhibition. This effect was not specific to +4 °C; similar behavior was observed at higher incubation temperatures (+10 °C and +25 °C). At 90 and 120 min, the induction effect was observed only at the farther-located bioreporters, 15 (Figure 5(b3,c3)) and 20 (Figure 5(b4,c4)) cm from the infection source. The possible reason for the observed induction effect at greater distances could be the diffusion patterns of the target VOCs. These compounds may accumulate more at farther distances, producing a stronger effect on the target bioreporters. This is evident in the increasing inhibition effect on the bioreporters located 15 and 20 cm away during the measurement period at +4 °C. A clear effect of incubation temperature on bacterial response can also be observed, with higher temperatures reducing response efficiency. By 120 min, no response was detected from cells at +25 °C, while bacteria at +10 °C still responded to the infection presence at the farthest distances (Figure 5(c1–c4)). The sensor demonstrated strong functionality in detecting VOCs associated with infections, particularly at lower temperatures, making it suitable for use in environments like storage rooms where produce is kept cool. However, the efficiency of the bacterial response decreases as the temperature increases, with higher temperatures leading to diminished sensor performance over time. This highlights the importance of maintaining optimal temperature conditions to ensure reliable sensor activity and effective monitoring of stored produce quality.

### 3.6. Sensitivity Assessment of the Bioreporter System

**Figure 6:**
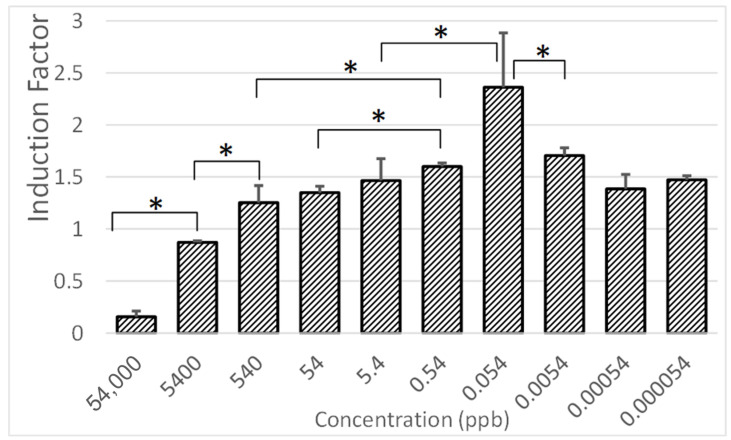
Bioluminescent response of the *E. coli DnaK* bioreporter to the different concentrations of 2-phenylethyl alcohol. Statistical analysis was performed using a paired two-sample *t*-test ((*) *p* < 0.05), with significant differences indicated by asterisks.

The system’s sensitivity was tested using a range of 2-Phenylethyl Alcohol (2-PEA) concentrations. In this study, ethanol was used as a general stress inducer and a positive control due to its well-documented impact on bacterial viability and stress-response gene expression. In contrast, 2-phenylethyl alcohol (2-PEA) was selected as a pathogen-specific biomarker, previously identified through GC-MS analysis as being emitted from infected potato tubers [9]. This makes 2-PEA a relevant biomarker for early disease detection in postharvest crops. To evaluate the system’s sensitivity to 2-PEA, a genetically engineered *E. coli* strain was used, in which the *DnaK* promoter was fused to a lux-based bioluminescent reporter plasmid, a system previously demonstrated to detect potato infections effectively [10]. The results in Figure 6 show that the bioluminescent response was concentration-dependent, reaching its peak at 0.054 ppb, indicating the system’s maximum sensitivity within this range. At the highest tested concentrations (54,000–540 ppb), an inhibition effect was observed, likely due to excessive molecular stress overwhelming the *DnaK*-mediated proteostasis system [28]. The relatively stable response in the mid-range concentrations (540–0.54 ppb) may be attributed to the balance between induction and inhibition, whereby 2-PEA simultaneously triggers the cell’s response while impairing cellular function at elevated levels. These findings provide insights into the sensitivity and dynamic range of the proposed biosensor system. The lower limit of detection (LOD) of 0.054 ppb suggests that the system can detect extremely low levels of 2-PEA, supporting its feasibility for the real-time monitoring of early infection stages. The bioluminescent response curve indicates a nonlinear relationship, which aligns with previous studies using whole-cell bacterial biosensors, whereby excessive stressors often induce toxicity rather than continuous induction [25]. What is interesting is the similarity in the system responses to 54 ppb and 0.00054 ppb. Although Figure 6 presents only the ratio between positive (ethanol-exposed) and negative (control) responses, expressed as the induction factor (IF), it does not reflect the full kinetic behavior of the response curves. In practice, the responses to 54 ppb and 0.00054 ppb were distinguished by differences in both the timing and intensity of bioluminescence: lower concentrations produced delayed yet measurable signals, while higher concentrations often triggered earlier responses or, at the highest levels, inhibition due to cellular stress. The observed response pattern suggests that the sensor’s optimal detection window lies within the 540–0.054 ppb range, making it suitable for detecting VOC emissions under realistic storage conditions.

### 3.7. Evaluation of Whole-Cell-Based Sensors’ Capacity for Real-Time and Continuous Monitoring of Pectobacterium Infection in Potato Tubers

**Figure 7:**
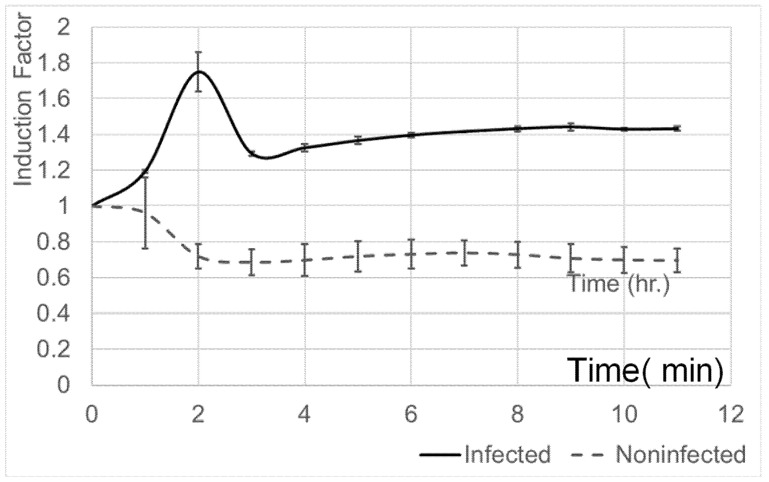
Responses of the whole-cell-based sensor coupled with an immobilized *DnaK* strain to potato tubers infected and not infected with *Pectobacterium brasiliense*.

To evaluate the biosensor’s ability to detect *Pectobacterium brasiliense* in potato tubers, the immobilized *E. coli DnaK* strain was placed near infected and non-infected tubers stored at +4 °C. As shown in Figure 6, the system effectively detected infection, with bioluminescence induced by volatiles from infected tubers, while slight inhibition was observed in response to non-infected samples, likely due to temperature effects. The *DnaK* strain, like TV1061 (Figure 7), showed peak induction two hours after exposure. This whole-cell biosensor enables real-time, continuous monitoring, which is crucial for early infection detection in stored produce. Its integration with a CMOS sensor enhances specificity by detecting disease-associated VOCs, making it highly accurate for postharvest applications. The system’s functionality at low temperatures preserves bacterial viability, ensuring reliability in cold storage conditions. While this study confirms its ability to identify infected tubers in real storage settings, further research is needed to determine the limit of detection (LOD), sensor linearity, and response dynamics to various VOC compositions. Expanding this approach with additional disease-specific strains could allow for the simultaneous detection of multiple pathogens, transforming postharvest management by enabling broad-spectrum monitoring and improving food safety and shelf life.

### 3.8. Summary and Future Directions

Determining optimal construction and operation conditions for biosensors is critical for their effectiveness in real-world applications, particularly for monitoring postharvest produce quality in storage environments. This study highlights the influence of various factors, such as polymerization processes, nutrient sources, and temperature, on sensor functionality. Ensuring uniform bacterial distribution within calcium alginate matrices and maintaining bacterial viability through a controlled nutrient supply is essential for consistent sensor performance. The findings demonstrate the sensor’s potential for use in low-temperature storage environments, crucial for extending the shelf life of perishable goods. As a proof of concept, the current study has successfully demonstrated the ability of immobilized whole-cell biosensors to detect infection-related VOCs in a real-world-like storage setting. However, further steps are necessary to advance this technology toward practical deployment.

Future developments will focus on thoroughly evaluating the system’s sensitivity, specificity, and dynamic range, particularly by quantifying its limit of detection (LOD) using pure volatile compounds and naturally infected samples at different infection stages. Moreover, improving the durability and operational stability of the biosensor under fluctuating environmental conditions will be a priority. To further enhance reliability, strategies such as incorporating more sophisticated nutrient delivery systems or engineering new immobilization materials that better preserve bacterial viability over extended periods will be explored. Expanding the range of detectable pathogens through the development of additional disease-specific bioreporter strains is another critical future direction, with the aim of achieving broad-spectrum, multiplexed detection capabilities. Integrating the biosensor platform with IoT technologies for automated, remote, and real-time monitoring will also be pursued to enable large-scale storage surveillance. Overall, continuous research and iterative optimization are required to refine these biosensor systems, making them highly adaptable, sensitive, and commercially viable tools for agriculture, food safety, and environmental monitoring.

## 4. Conclusions

In this study, we establish a proof of concept for a real-time, whole-cell-based biosensor capable of detecting early-stage crop infections through VOC sensing under cold storage conditions. The uniformity of bacterial distribution, the choice of nutrient sources, and the operating temperature were identified as key parameters affecting sensor performance. Our findings underscore the importance of optimizing these conditions to ensure reliable and accurate sensor functionality, especially in low-temperature storage environments. Additionally, we provide proof-of-concept capabilities for detecting infection in real storage rooms at varying temperatures using portable whole-cell-based applications. Moving forward, enhancing sensor durability, expanding analyte detection capabilities, and integrating real-time monitoring technologies will be essential. Continued innovation in this field will advance the practical application of biosensors in agriculture, food safety, and environmental monitoring, providing more effective tools for quality control and safety assurance.

## Figures and Tables

**Table 1:** Composition of nutrient media and their effects on bacterial growth and bioluminescence response.

Medium	Key Components	Growth Rate	Bioluminescence (RLUs)	Stability Over Time	Notes
LB	Tryptone, Yeast Extract, and NaCl	Moderate	Stable and selective	Long-term viability	The best balance for biosensor applications
SOC	Tryptone, Yeast Extract, Glucose, MgCl_2_, and MgSO_4_	High (initially)	Rapid decline	Short-lived stability	Likely osmotic stress effects
Terrific Broth	Tryptone, Yeast Extract, Glycerol, and Phosphate buffer	Highest	Unwanted background luminescence	Unstable	Glycerol induces non-specific bioluminescence
Tryptone	Tryptone only	Low	Weak response	Limited viability	Lacks essential cofactors for stable metabolism
Yeast Extract	Yeast Extract only	Low	Delayed induction	Short-term stability	Nutrient-limited growth
Minimal Salt	Inorganic salts, with no carbon source	No growth	No response	Not viable	Insufficient for bacterial maintenance

## Data Availability

The data presented in this study are available on request from the corresponding author. The data are not publicly available due to ongoing related research and confidentiality considerations.

## Supplementary Materials

The following supporting information can be downloaded at: https://www.mdpi.com/article/10.3390/bios15050300/s1, Figure S1: Schematic illustration of the bioluminescent biosensor system for VOC detection in stored potatoes.
