# Supplementary material for: Optimizing Whole-Cell Biosensors for the Early Detection of Crop Infections: A Proof-of-Concept Study
Source: Biosensors (Basel). 2025 May 8;15(5):300. doi: 10.3390/bios15050300 (PMC12109988; doi:10.3390/bios15050300)
Supplement: Supplementary file 1 [file biosensors-15-00300-s001.zip › biosensors-3538833-supplementary.pdf]

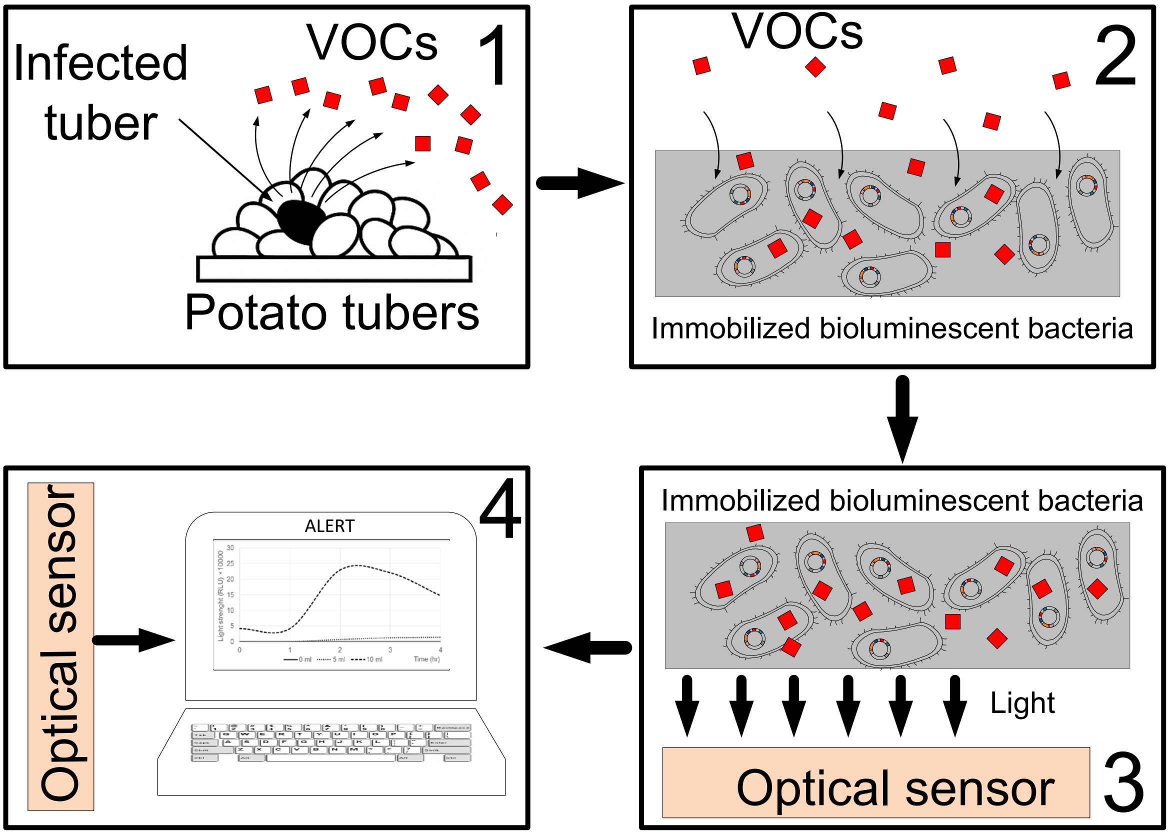

Figure S1: Schematic illustration of the bioluminescent biosensor system for VOC detection in stored potatoes. (1) Volatile organic compounds (VOCs) are emitted from infected potato tubers during storage. (2) These VOCs diffuse and interact with immobilized bioluminescent bacteria within a hydrogel matrix. (3) In response to specific VOCs, the engineered bacteria emit light, which is detected by an optical sensor placed beneath the tablets. (4) The sensor's output is processed by a computer, triggering an alert when light intensity exceeds a predefined threshold, indicating early signs of crop infection.
